# High‐Pressure Synthesis of Metal–Inorganic Frameworks Hf_4_N_20_⋅N_2_, WN_8_⋅N_2_, and Os_5_N_28_⋅3 N_2_ with Polymeric Nitrogen Linkers

**DOI:** 10.1002/anie.202002487

**Published:** 2020-05-08

**Authors:** Maxim Bykov, Stella Chariton, Elena Bykova, Saiana Khandarkhaeva, Timofey Fedotenko, Alena V. Ponomareva, Johan Tidholm, Ferenc Tasnádi, Igor A. Abrikosov, Pavel Sedmak, Vitali Prakapenka, Michael Hanfland, Hanns‐Peter Liermann, Mohammad Mahmood, Alexander F. Goncharov, Natalia Dubrovinskaia, Leonid Dubrovinsky

**Affiliations:** ^1^ Department of Mathematics Howard University 2400 Sixth Street NW Washington DC 20059 USA; ^2^ Bayerisches Geoinstitut University of Bayreuth Universitätstrasse 30 95440 Bayreuth Germany; ^3^ The Earth and Planets Laboratory Carnegie Institution for Science 5241 Broad Branch Road, NW Washington DC 20015 USA; ^4^ Center for Advanced Radiation Sources University of Chicago 9700 South Cass Avenue Lemont IL 60437 USA; ^5^ Material Physics and Technology at Extreme Conditions Laboratory of Crystallography University of Bayreuth Universitätstrasse 30 95440 Bayreuth Germany; ^6^ Materials Modeling and Development Laboratory National University of Science and Technology “MISIS” 119049 Moscow Russia; ^7^ Department of Physics, Chemistry and Biology (IFM) Linköping University 58183 Linköping Sweden; ^8^ European Synchrotron Radiation Facility BP 220 38043 Grenoble Cedex France; ^9^ Photon Science, Deutsches Elektronen-Synchrotron Notkestrasse 85 22607 Hamburg Germany

**Keywords:** high-pressure synthesis, inclusion compounds, inorganic double helix, metal–inorganic frameworks, polynitrides

## Abstract

Polynitrides are intrinsically thermodynamically unstable at ambient conditions and require peculiar synthetic approaches. Now, a one‐step synthesis of metal–inorganic frameworks Hf_4_N_20_⋅N_2_, WN_8_⋅N_2_, and Os_5_N_28_⋅3 N_2_ via direct reactions between elements in a diamond anvil cell at pressures exceeding 100 GPa is reported. The porous frameworks (Hf_4_N_20_, WN_8_, and Os_5_N_28_) are built from transition‐metal atoms linked either by polymeric polydiazenediyl (polyacetylene‐like) nitrogen chains or through dinitrogen units. Triply bound dinitrogen molecules occupy channels of these frameworks. Owing to conjugated polydiazenediyl chains, these compounds exhibit metallic properties. The high‐pressure reaction between Hf and N_2_ also leads to a non‐centrosymmetric polynitride Hf_2_N_11_ that features double‐helix catena‐poly[tetraz‐1‐ene‐1,4‐diyl] nitrogen chains [−N−N−N=N−]_∞_.

## Graphical abstract

**A one‐step high‐pressure** synthetic route is described to complex metal–inorganic frameworks built of transition metals (Hf, Os and W) that are connected via polymeric nitrogen linkers. The pores are filled with ordered one‐dimensional arrays of nitrogen molecules. Owing to the conjugated π‐systems, these inorganic polymers are conductive materials.

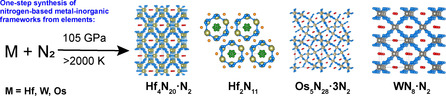

Homoatomic bonding, being a prominent feature of carbon chemistry, is also characteristic for pnictogens, which readily form extended polymers and homoatomic frameworks.^1^, ^2^ Contrary to carbon and phosphorus, the nitrogen–nitrogen single and double bonds possess much less than 1/3 and 2/3, respectively, of the energy of the triple bond. Therefore, the conversion of N−N single or double bonds to triple bonds results in a very large energy release and makes the polynitrides highly endothermic and thermodynamically unstable at atmospheric pressure. At high pressures, however, the decomposition of such phases with the evolution of nitrogen is efficiently suppressed that provides a playground for systematic studies of nitrogen‐rich compounds, in which various nitrogen‐containing polyanions can be stabilized by simple metal cations. Even if such compounds may appear to be unstable at ambient conditions, high‐pressure experiments provide a proof of their existence and valuable information for further development of the ambient‐pressure synthesis of nitrogen‐rich phases. So, the first successful synthesis of alkali metal pentazolates (salts containing *cyclo*‐N_5_^−^ anions) was performed at high pressure.^3^ Later, unsubstituted *cyclo*‐N_5_^−^ was stabilized at ambient conditions too.^4^, ^5^, ^6^, ^7^, ^8^ Metal–pentazolate frameworks (AgN_5_, Cu(N_5_)(N_3_),^6^, ^9^ Na_24_N_60_, Na_20_N_60_ 5) might not only have applications as energetic materials, but also be intrinsically interesting as direct inorganic structural analogues of azolate metal–organic frameworks and framework materials based on an aromatic inorganic linker.^9^, ^10^ Metal–azide frameworks based on transition metals linked through bridging azido ligands are important for the development of molecular magnets.^11^, ^12^

Recent high‐pressure studies have shown that various polynitrogen species may occur as a result of simple reaction between a metal and molecular nitrogen. Good examples of that are FeN_4_ containing catena‐poly[tetraz‐1‐ene‐1,4‐diyl] anions [−N=N−N−N−]^2−^_∞_,^13^, ^14^ MgN_4_, and ReN_8_⋅N_2_ with polydiazenediyl (polyacetylene‐like) nitrogen chains.^15^, ^16^ Thus, high‐pressure synthesis conditions enable the exploration of numerous possible metal–nitrogen framework topologies.

Herein we studied chemical reactions between 5d transition metals Hf, W, Os, and nitrogen in laser‐heated diamond anvil cells at pressures exceeding 1 Mbar. All metals were found to form metal–inorganic frameworks MIFs: Hf_4_N_20_⋅N_2_ (**1**), WN_8_⋅N_2_ (**2**), Os_5_N_28_⋅3 N_2_ (**3**) with polymeric nitrogen linkers and guest dinitrogen molecules that are arranged in one‐dimensional arrays. In all experiments discussed herein, a piece of metal was placed inside a sample chamber in a BX90 diamond anvil cell^17^ loaded with nitrogen that served as a reagent and as a pressure‐transmitting medium. Hf, W, and Os samples were compressed to a pressure of about 105 GPa and laser‐heated to 1900(200), 2700(200), and 2800(150) K, respectively. The reaction products contained multiple good‐quality single‐crystalline domains of novel phases, which were studied using synchrotron single‐crystal X‐ray diffraction (SCXRD) at the beamlines P02.2 (PetraIII, DESY, Hf sample), GSECARS 13IDD (APS, W and Hf samples), ID15b (ESRF, W sample) and ID11 (ESRF, Os sample). More details on the experimental procedures are given in the Supporting Information.

The crystal‐structure solution and refinement revealed the chemical formulas of new compounds as Hf_4_N_20_⋅N_2_, WN_8_⋅N_2_ and Os_5_N_28_⋅3 N_2_ (Figure 1)_._ The refinement against SCXRD data resulted in very good agreement factors (Supporting Information, Table S1). For a cross‐validation of the crystal structures we performed theoretical calculations based on density functional theory. We carried out the full structure optimization for all of the compounds from ambient to the synthesis pressure and found that optimized crystal structures are in a very good agreement with the experimental ones (Supporting Information, Table S2).

**Figure 1:**
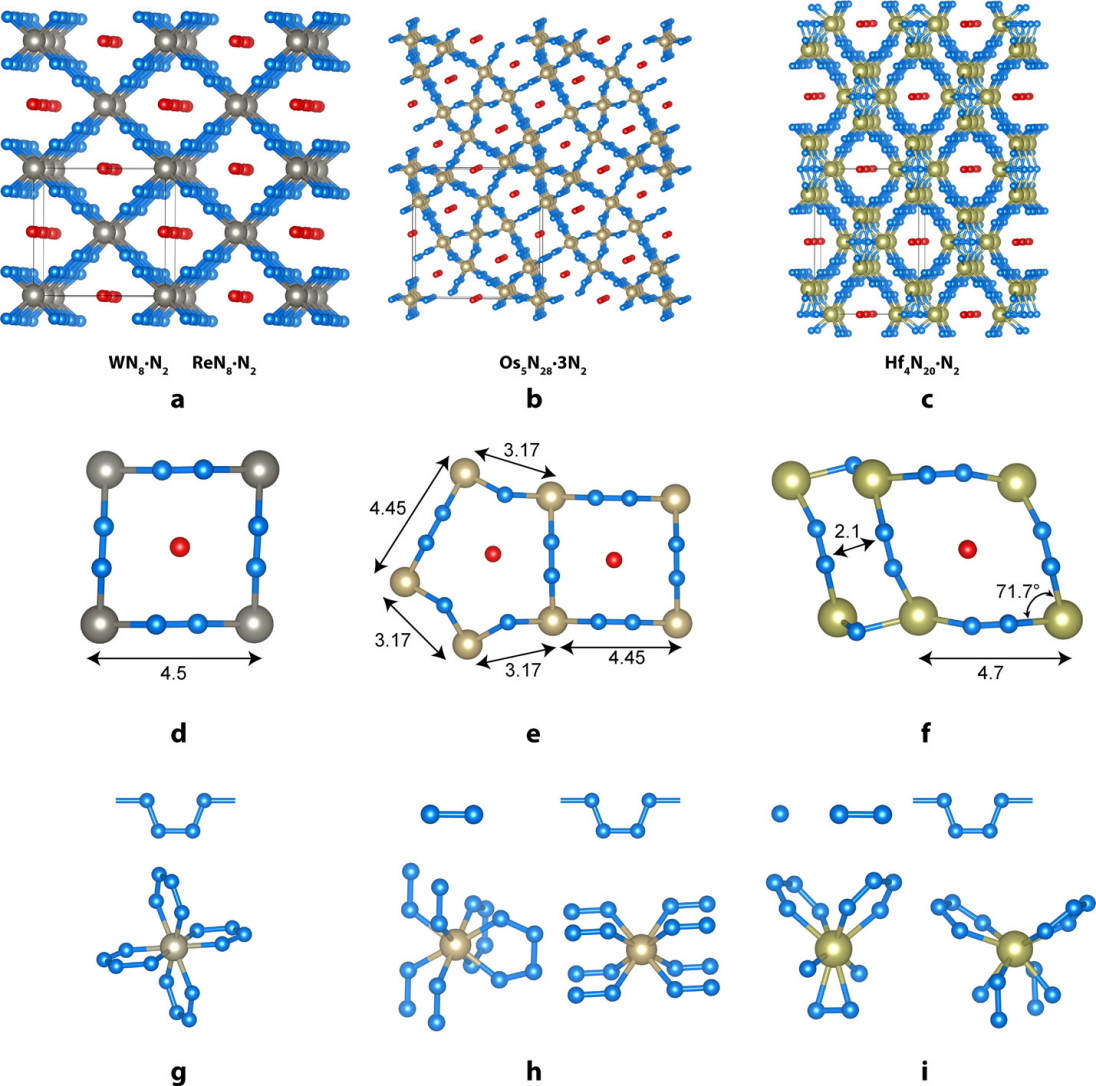
a)–f) Crystal structures of a) WN_8_⋅N_2_, b) Os_5_N_28_⋅3 N_2_, and c) Hf_4_N_20_⋅N_2_, and geometries of the channels in the crystal structures (d–f). Distances are given in Å. g)–i) Types of nitrogen units building the network and types of metal coordination in corresponding compounds. Blue spheres represent nitrogen atoms that are the part of the framework. Red spheres are dinitrogen molecules confined in the channels. Larger spheres represent corresponding transition metals.

The structure of WN_8_⋅N_2_ has the orthorhombic space group *Immm* with one W and two N atomic positions. WN_8_⋅N_2_ is isostructural to previously reported inclusion compound ReN_8_⋅N_2_.^15^ W atoms are eightfold coordinated by four planar polydiazenediyl nitrogen chains with conjugated π‐systems (Figure 1 g, Scheme 1). The 3D framework WN_8_ possesses rectangular‐shaped channels that are occupied by dinitrogen molecules (Figure 1 d). In the ionic consideration, each W atom gives four of its six valence electrons to four N_4_ units. Thus, each N_4_ unit accommodates 2 electrons and has 22 valence electrons in total: four N−N σ bond pairs (8*e*), four dative N→W bonds (8*e*), and six π‐delocalized electrons with an effective N−N bond order of 1.25. Tungsten, therefore, should have a formal oxidation state +IV and the formula of the framework can be written as W^4+^(N_4_^2−^)_2_. The framework topology can be described by a point symbol {5^2^.6}_8_{5^4^.6^12^.9^12^} as determined by the software ToposPro.^18^

**Scheme 1:**
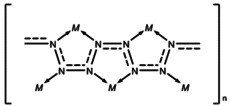
Coordination of metal atoms by a polydiazenediyl (polyacetylene‐like) nitrogen chain.

The structure of Os_5_N_28_⋅3 N_2_ has the orthorhombic space group *Pnnm* with 3 Os and 9 N atomic positions. It exhibits two types of nitrogen units: N−N dumbbells and polydiazenediyl chains, so that the chemical formula of the framework may be rewritten as Os_5_(N_2_)_6_(N_4_)_4_. The nitrogen–nitrogen distances within the dumbbells vary in a range 1.22–1.33 Å, which is characteristic for a double bond of the [N=N]^2−^ unit. This is in a good agreement with the common oxidation state of osmium +IV and the formula of the framework can be written as (Os^4+^)_5_(N_2_^2−^)_6_(N_4_^2−^)_4_. All Os atoms possess distorted cubic coordination by nitrogen atoms. Os1 atoms are coordinated only by nitrogen atoms that form dumbbells, while Os2 and Os3 atoms are coordinated by both type of units in the proportion 1:1 (Figure 1 h). The framework contains rectangular and octagonal‐shaped channels occupied by dinitrogen molecules. The framework topology can be described by a point symbol {4.6^2^}_12_{4^2^.5^2^.6^14^.8^8^.9^2^}_4_{4^4^.6^16^.8^8^}{5^2^.6}_16_.

The structure of Hf_4_N_20_⋅N_2_ has the orthorhombic space group *Cmmm* with 2 Hf and 5 N atomic positions. Among all reported compounds, Hf_4_N_20_⋅N_2_ has the highest number of various nitrogen units: polydiazenediyl nitrogen chains, N−N dumbbells, and discrete N atoms. Nitrogen–nitrogen distances within the dumbbells are 1.32 Å and, therefore, the formula can be written as (Hf^4+^)_4_(N^3−^)_2_(N_2_^2−^)(N_4_^2−^)_4_ with Hf^4+^ in good agreement with the most common oxidation state of Hf (+IV). The framework contains rhombus‐shaped channels occupied by dinitrogen molecules. The framework topology can be described by the point symbol {3.4^4^.5^6^.6^3^.7^8^.8^4^.9^2^}{3^2^.4^5^.5^3^}{4^5^.5^6^.6^3^.7^8^.8^4^.9^2^}{4^5^.5}{5^3^}_8_.

The frameworks of all of the compounds **1**–**3** and ReN_8_⋅N_2_ 15 are built of a set of standard units: N−N dumbbells, discrete N^3−^ anions and polydiazenediyl chains. An important factor for the stability of the polydiazenediyl chains is a resonance, which imparts partial double‐bond character for nitrogen–nitrogen bonds. The stabilization through the resonance is well‐known, with the classic examples of azides and pentazolates.^19^ Recently synthesized MgN_4_ featuring polydiazenediyl chains could be even recovered at ambient conditions.^16^ Polydiazenediyl chains are also theoretically predicted to exist in CaN_4_, ReN_4_, and HfN_8_⋅N_2_.^20^, ^21^, ^22^, ^23^ Along with a pentazolate anion, the polydiazenediyl chain may be one of the most stable structural units to be preserved at ambient pressures.

The compounds **1**–**3** can be rationalized based on the Mooser–Pearson extended (8−*N*) rule.^24^ For the polynitride compound MN*x*, the number of nitrogen–nitrogen bonds per one nitrogen atom, *b*(NN), can be calculated as *b*(NN)=(8−[*e*(M)+*xe*(N)]/*x*), where *e*(M) and *e*(N) are the numbers of valence electrons of metal and nitrogen, respectively. The results of the calculations applied to polynitrides are presented in the Table 1. It should be noted that *b*(NN) is fully consistent with the assignments of the bond orders from the crystal‐chemical analysis and with the calculated charge density maps (discussed below).

**Table 1:** Application of the Mooser–Pearson extended (8−*N*) rule for the description of polynitrides.

Unit	Bonds/atom	Number of atoms belonging to the unit		
		Hf_4_N_20_⋅N_2_	WN_8_⋅N_2_	Os_5_N_28_⋅3 N_2_
N≡N	3	2	2	6
[N_4_]_∞_^2−^ Polydiazenediyl	2.5	16	8	16
[N=N]^2−^	2	2	0	12
[N‐N]^4−^	1	0	0	0
N^3−^	0	2	0	0
Av. no. of covalent N−N bonds per N atom (crystal‐chemical analysis)		2.27	2.6	2.41
Av. no. of covalent N−N bonds per N atom (the (8−*N*) rule)		2.27	2.6	2.41

To gain a deeper insight into the electronic properties of the compounds, we calculated the electronic density of states (DOS) and the electron localization function (ELF). At high pressure, all the considered materials are metallic and the main contribution to the DOS at the Fermi level comes from nitrogen chains (Figure 2) forming delocalized π‐bonds, as demonstrated by the spatial distribution of electronic density in the range −0.1:0 eV (the inset in Figure 2 b shows an example of WN_8_⋅N_2_).

**Figure 2:**
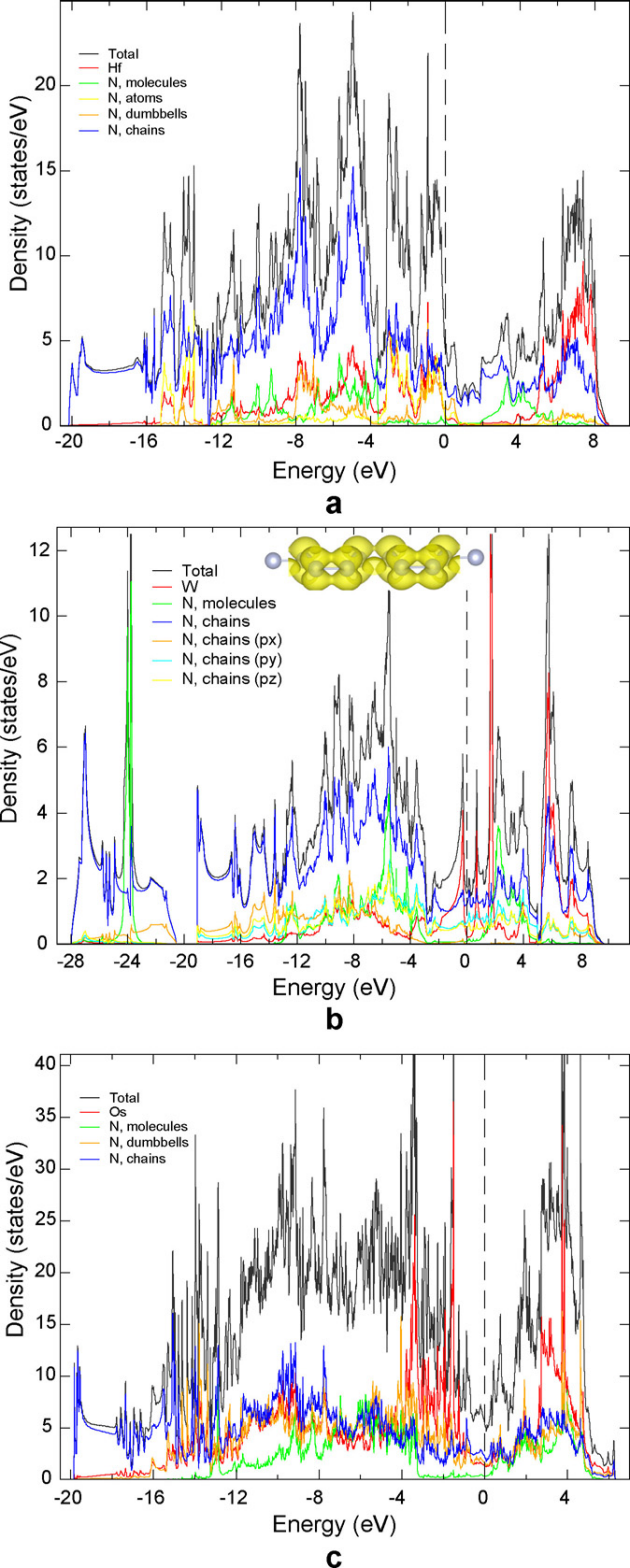
Calculated electronic densities of states for a) Hf_4_N_20_⋅N_2_ at *P*=96.4 GPa, b) WN_8_⋅N_2_ at *P*=103 GPa, and c) Os_5_N_28_⋅3 N_2_ at *P*=108 GPa. The inset in (b) shows a spatial distribution of the electronic density in the range −0.1:0 eV on the N atoms of the chains.

The ELF demonstrates strong covalent bonding between nitrogen atoms: the attractor associated with the N−N bond is located halfway between the atomic spheres (Figure 3 a,c,e). The attractor thickness along the N−N bonds decreases from the nitrogen chains to dumbbells, and to nitrogen molecules. This demonstrates the increase of the bond order (from about 1.25 to 2, and to 3, respectively) that is in a good agreement with the crystal‐chemical analysis.

**Figure 3:**
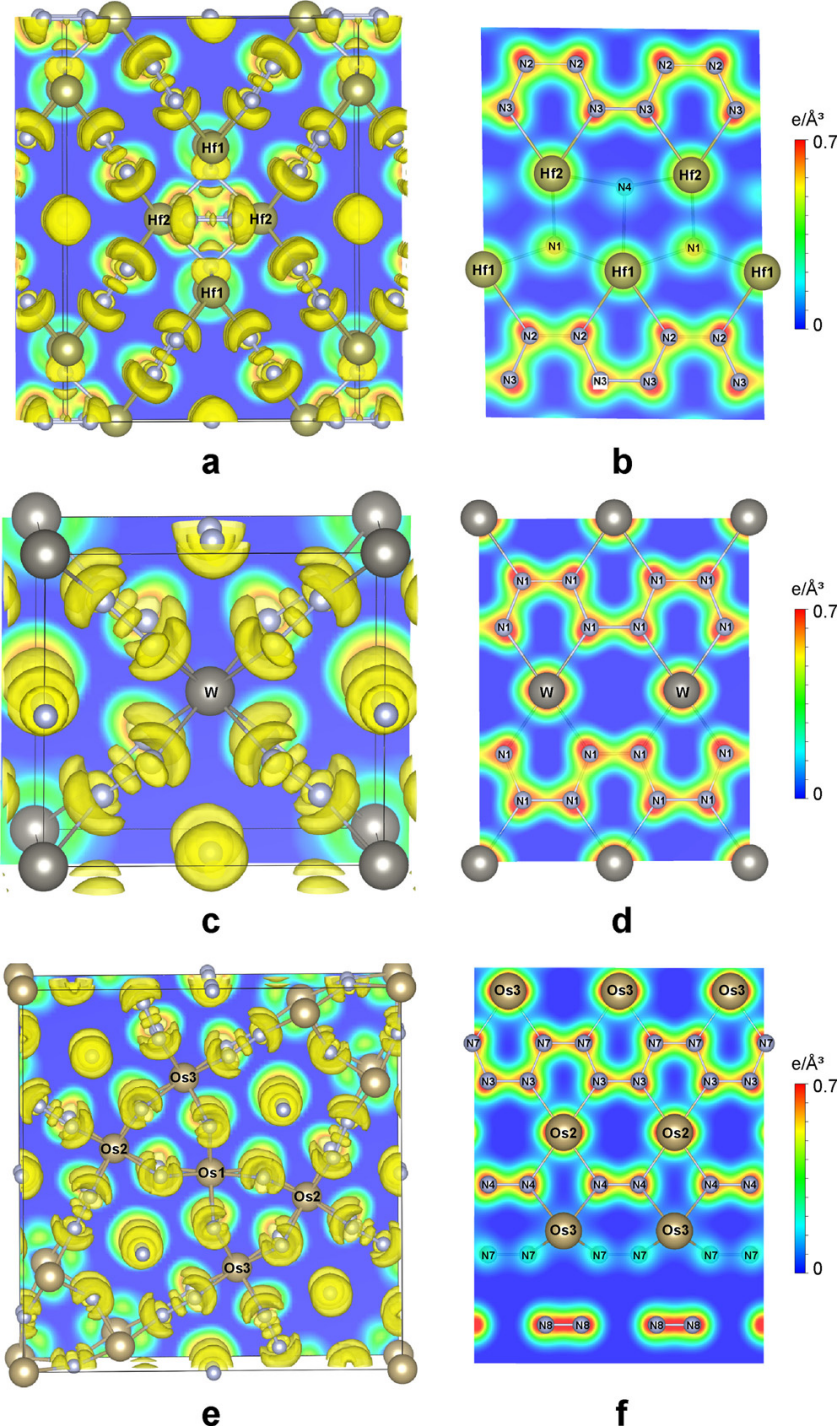
Calculated electron localization function with isosurface value 0.8 (a,c,e) and charge density maps (b,d,f) for Hf_4_N_20_⋅N_2_ (a,b) at *P*=96.4 GPa, WN_8_⋅N_2_ (c,d) at *P*=103 GPa, and Os_5_N_28_⋅3 N_2_ (e,f) at *P*=108 GPa.

Metal–nitrogen bonds reveal a different type of the ELF maxima. Separated attractors shifted toward the nitrogen atoms are found on the Me−N bond lines suggesting formation of two‐center polar covalent bonds. Both discrete nitrogen atoms and those of the dumbbell form three‐center Me−N−Me bonds with Os and Hf atoms with slight delocalization. According to the calculated charge density maps (Figure 3 b,d,f), the electron density is nearly similar between all of the atoms of the nitrogen chains with only a slight increase on the bonds parallel to the line connecting the metal atoms compared to the side bonds. This leads to a small difference in the lengths of N−N bonds in polydiazenediyl chains.

Although the synthesis of some porous materials may require high pressure (for example, ZIF‐8,^25^ hydrothermal synthesis of zeolites^26^), the formation of porous structures at megabar pressures seems to be counterintuitive, as these conditions are expected to destabilize less dense framework structures. However, the pressures of about 110 GPa and temperatures of 2000 K are the conditions of the thermodynamic equilibrium between molecular and polymeric nitrogen, thus the volume gain of polymeric nitrogen is balanced by the energy of the triple nitrogen–nitrogen bond.^27^ Furthermore, the polar covalent character of M−N bonds means that polydiazenediyl chains act as classical ligands (as donors for N−Me bonding). This imposes additional restrictions on the geometry of the system, namely, because of the sp^2^ hybridization of nitrogen atoms, a metal must be in the same plane as the coordinating nitrogen chain. It is indeed the case for the compounds **1**–**3** studied in the current work and ReN_8_⋅N_2_,^15^ which enables us to suggest that the covalent character of the transition metal–nitrogen bonds is one of the important factors for the framework architecture: the imposed geometry constraints lead to the formation of framework structures, rather than to the densest packing of atoms even at ultrahigh pressures. Another factor stabilizing the compounds **1**–**3** is the inclusion of nitrogen molecules into the pores. Entrapment of a pressure‐transmitting medium by metal–organic frameworks upon compression is a well‐known phenomenon, which significantly affects their properties, such as compressibility, amorphization pressures, and the sequence of phase transitions.^28^, ^29^, ^30^

The synthesis of nitrogen‐rich MIFs requires a substantial excess of nitrogen over metal. Owing to the design of the DAC experiment, in which the reagents are initially in different states of aggregation (metal is solid, and nitrogen is gaseous), it is not possible to make a homogenous mixture with a definite M:N ratio. Therefore, the formation of MIFs happens at the interface between a metal piece and nitrogen (Figure 4), whereas the bulk of the metal sample either remains intact or partially transforms to other nitrides. For example, a considerable part of the Os sample (Figure 4 c) turned into osmium pernitride OsN_2_ with the marcasite structure type.^31^

**Figure 4:**
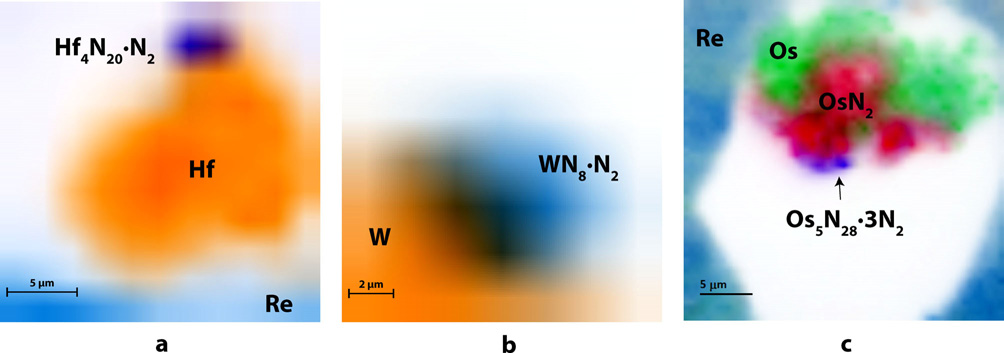
X‐ray diffraction imaging maps of samples containing a) Hf_4_N_20_⋅N_2_, b) WN_8_⋅N_2_, and c) Os_5_N_28_⋅3 N_2_.

In the Hf–N system, the reaction product, along with Hf_4_N_20_⋅N_2_, contains a novel compound with a chemical formula Hf_2_N_11_ (**4**) (Figure 5; Supporting Information, Tables S3, S4). The structure of Hf_2_N_11_ is built of Hf atoms coordinated by discrete nitrogen atoms, N=N dumbbells and catena‐poly[*trans*‐tetraz‐1‐ene‐1,4‐diyl] chains [−N=N−N−N−] (Figure 5 c) similar to those previously observed in FeN_4_.^13^ Discrete nitrogen atoms and dumbbells are within Hf_4_ tetrahedra (Figure 5 d), while polymeric nitrogen chains form double‐helix structure (Figure 5 e). Inorganic double helix structures are extremely rare and may have extraordinary properties.^32^, ^33^ The crystal chemical formula of Hf_2_N_11_ may be written as Hf_2_N(N_4_)_2_(N_2_). It should be noted that the charge balance is not achieved if the N_2_ dumbbell unit has a charge of −2 or −4. The N−N distance within this unit is 1.186 Å, which is significantly shorter than in [N=N]^2−^ or [N−N]^4−^. We, therefore, can suggest that Hf_2_N_11_ contains N_2_^−^ units with an effective N−N bond order of 2.5. This agrees with the charge balance (Hf^4+^)_2_N^3−^(N_4_^2−^)_2_(N_2_^−^) and with the 8−*N* rule. Such N_2_^−^ units were recently reported in CuN_2_ at high pressure.^34^

**Figure 5:**
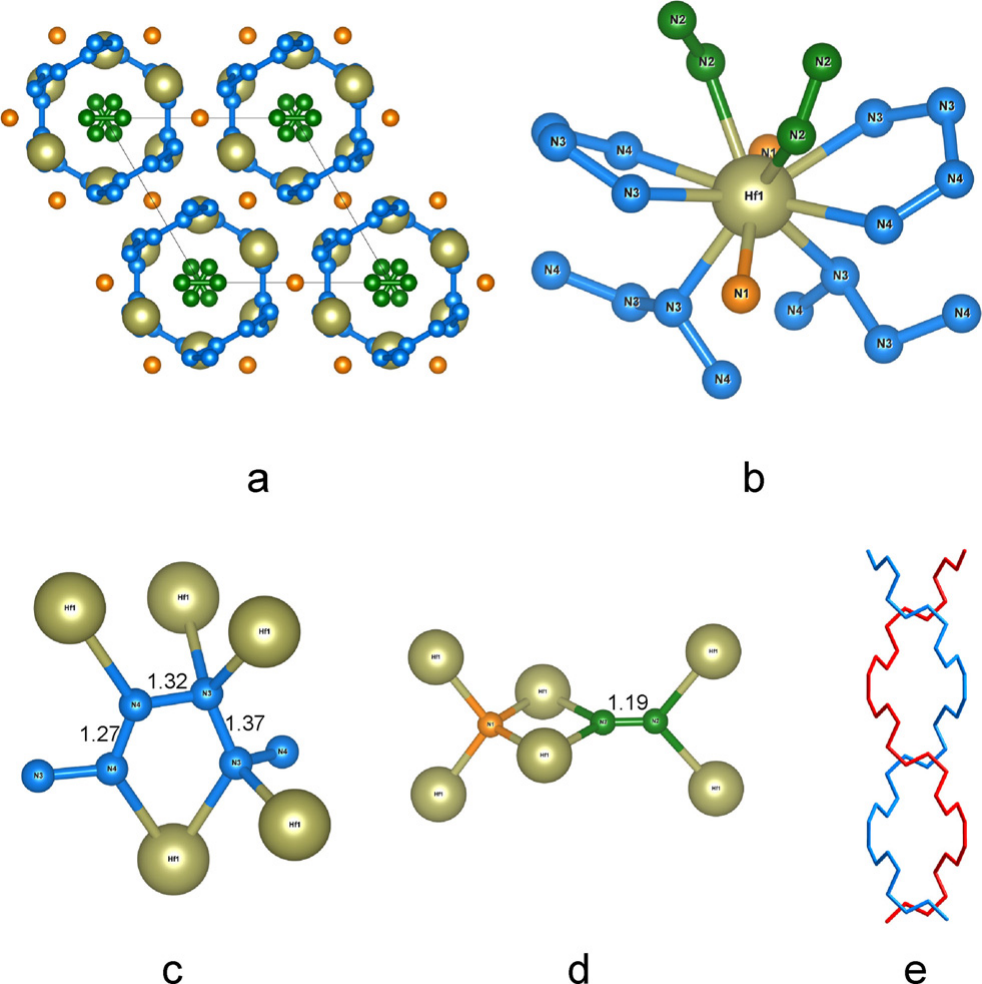
The crystal structure of Hf_2_N_11_. a) View of the crystal structure along the *c*‐axis. Blue: N atoms of the infinite chains, green: N atoms that form dumbbells, orange: discrete N atoms. b) Coordination environment of the Hf atom. c) Coordination environment of polymeric nitrogen chains. d) Coordination environment of discrete nitrogen atoms and nitrogen dumbbells. e) Double‐helix chain built of nitrogen atoms running along the *c*‐direction.

The framework HfN_8_⋅N_2_, isostructural to WN_8_⋅N_2_ and ReN_8_⋅N_2_, was previously predicted by Zhang et al.^22^ The absence of this topology among the Hf compounds we observed in our experiments may have the following explanation. First, whereas WN_8_ obeys the 18*e* rule and ReN_8_, as a 19*e* complex, shows just a small deviation, the HfN_8_ framework would have been a less stable 16*e* complex (the same reasoning could also explain why Os does not form a 20*e* OsN_8_ complex). Second, Hf metal has much lower electronegativity and readily forms bonds of more ionic character that explains N^3−^ and N_2_^2−^ ions in the crystal structure of Hf_4_N_20_⋅N_2_ and Hf_2_N_11_.

Phonon dispersion relations calculated for compounds **2**–**4** show their dynamic stability at high pressure, as evidenced by the absence of any imaginary phonon modes (Supporting Information, Figures S1–S3). At the same time, the phonon dispersion curves of WN_8_⋅N_2_ (compound **2**) and Hf_2_N_11_ (**4**) do have imaginary modes at atmospheric pressure that suggests their instability at ambient pressure and *T*=0 K. Indeed, WN_8⋅_N_2_ produced good diffraction patterns down to about 25 GPa. Further pressure release resulted in an unknown compound with the following lattice parameters: *a*=5.431(5), *b*=6.36(7), *c*=7.236(9) Å, *α*=*γ*=90°, *β*=95.3(1)°. Unfortunately, the quality of its diffraction pattern did not allow a reliable structure solution. We would like to note that small magnitude of the imaginary frequencies seen in Hf_2_N_11_ and WN_8_⋅N_2_ at ambient pressure is expected to be removed at finite temperature by anharmonic effects (renormalization).^35^ Similar argument supports the stabilization of Hf_4_N_10_⋅N_2_ at finite temperature even though phonon calculations in the harmonic approximation at *T*=0 K resulted in small imaginary frequencies for some optical modes (Supporting Information, Figure S4).

The decompressed Os–N sample contained only Os and OsN_2_ (Supporting Information, Figure S5). The crystal quality of both Hf_2_N_11_ and Hf_4_N_20_⋅N_2_ deteriorated rapidly on decompression and the diffraction from these compounds was almost undetectable below 80 GPa. In the search of the predicted HfN_8_⋅N_2_ compound at lower pressures, we re‐heated the sample at 73 GPa.^22^ The resulting material was cubic Hf_3_N_4_ (*I* $\bar{4}$ 3*d*, No. 220, *a=*6.2946(13) Å), as evident from the SCXRD analysis (Supporting Information, Table S5).^36^

In conclusion, at extremely high pressures we have discovered three novel inclusion compounds, Hf_4_N_20_⋅N_2_, WN_8_⋅N_2_, and Os_5_N_28_⋅3 N_2_, which are built from host metal–inorganic frameworks and guest dinitrogen molecules. The one‐step synthesis of these materials is achieved via a reaction between elemental metal and nitrogen. Their common characteristic structural units, the resonance‐stabilized polydiazenediyl (polyacetylene‐like) chains, may appear to be appropriate building blocks of other nitrogen‐rich compounds that offer an elegant approach to the purposed synthesis of various metal–inorganic frameworks and enables further exploration of the remarkable chemistry of polynitrides.

## Conflict of interest

The authors declare no conflict of interest.

## Supporting information

As a service to our authors and readers, this journal provides supporting information supplied by the authors. Such materials are peer reviewed and may be re‐organized for online delivery, but are not copy‐edited or typeset. Technical support issues arising from supporting information (other than missing files) should be addressed to the authors.

Supplementary

## References

[1] H. G. Von Schnering , W. Hoenle , Chem. Rev. 1988, 88, 243–273.

[2] J. Wang , J.-A. Dolyniuk , K. Kovnir , Acc. Chem. Res. 2018, 51, 31–39. 29256588 10.1021/acs.accounts.7b00469

[3] B. A. Steele , E. Stavrou , J. C. Crowhurst , J. M. Zaug , V. B. Prakapenka , I. I. Oleynik , Chem. Mater. 2017, 29, 735–741.

[4] C. Zhang , C. Sun , B. Hu , C. Yu , M. Lu , Science 2017, 355, 374–376. 28126812 10.1126/science.aah3840

[5] W. Zhang , K. Wang , J. Li , Z. Lin , S. Song , S. Huang , Y. Liu , F. Nie , Q. Zhang , Angew. Chem. Int. Ed. 2018, 57, 2592–2595; 10.1002/anie.201710602 29336110

[6] C. Sun , C. Zhang , C. Jiang , C. Yang , Y. Du , Y. Zhao , B. Hu , Z. Zheng , K. O. Christe , Nat. Commun. 2018, 9, 1269. 29593262 10.1038/s41467-018-03678-y PMC5871778

[7] Y. Xu , Q. Wang , C. Shen , Q. Lin , P. Wang , M. Lu , Nature 2017, 549, 78–81. 28847006 10.1038/nature23662

[8] Y. Xu , L. Tian , D. Li , P. Wang , M. Lu , J. Mater. Chem. A 2019, 7, 12468–12479.

[9] Y. Xu , Q. Lin , P. Wang , M. Lu , Chem. Asian J. 2018, 13, 1669–1673. 29701898 10.1002/asia.201800476

[10] M. Arhangelskis , A. D. Katsenis , A. J. Morris , T. Friščić , Chem. Sci. 2018, 9, 3367–3375. 29780467 10.1039/c7sc05020h PMC5933226

[11] F. A. Mautner , R. Cortés , L. Lezama , T. Rojo , Angew. Chem. Int. Ed. Engl. 1996, 35, 78–80;

[12] M. A. S. Goher , J. Cano , Y. Journaux , M. A. M. Abu-Youssef , F. A. Mautner , A. Escuer , R. Vicente , Chem. Eur. J. 2000, 6, 778–784. 10826599 10.1002/(sici)1521-3765(20000303)6:5<778::aid-chem778>3.0.co;2-p

[13] M. Bykov , E. Bykova , G. Aprilis , K. Glazyrin , E. Koemets , I. Chuvashova , I. Kupenko , C. McCammon , M. Mezouar , V. Prakapenka , et al., Nat. Commun. 2018, 9, 2756. 30013071 10.1038/s41467-018-05143-2 PMC6048061

[14] M. Bykov , S. Khandarkhaeva , T. Fedotenko , P. Sedmak , N. Dubrovinskaia , L. Dubrovinsky , Acta Crystallogr. Sect. E 2018, 74, 1392–1395. 10.1107/S2056989018012161 PMC6176440 30319786

[15] M. Bykov , E. Bykova , E. Koemets , T. Fedotenko , G. Aprilis , K. Glazyrin , H.-P. Liermann , A. V. Ponomareva , J. Tidholm , F. Tasnádi , et al., Angew. Chem. Int. Ed. 2018, 57, 9048–9053; 10.1002/anie.201805152 29774981

[16] D. Laniel , B. Winkler , E. Koemets , T. Fedotenko , M. Bykov , E. Bykova , L. Dubrovinsky , N. Dubrovinskaia , Nat. Commun. 2019, 10, 4515. 31586062 10.1038/s41467-019-12530-w PMC6778147

[17] I. Kantor , V. Prakapenka , A. Kantor , P. Dera , A. Kurnosov , S. Sinogeikin , N. Dubrovinskaia , L. Dubrovinsky , Rev. Sci. Instrum. 2012, 83, 125102. 23278021 10.1063/1.4768541

[18] V. A. Blatov , A. P. Shevchenko , D. M. Proserpio , Cryst. Growth Des. 2014, 14, 3576–3586.

[19] K. O. Christe , Propellants Explos. Pyrotech. 2007, 32, 194–204.

[20] S. Yu , B. Huang , Q. Zeng , A. R. Oganov , L. Zhang , G. Frapper , J. Phys. Chem. C 2017, 121, 11037–11046.

[21] S. Zhu , F. Peng , H. Liu , A. Majumdar , T. Gao , Y. Yao , Inorg. Chem. 2016, 55, 7550–7555. 27428707 10.1021/acs.inorgchem.6b00948

[22] J. Zhang , A. R. Oganov , X. Li , H. Niu , Phys. Rev. B 2017, 95, 020103.

[23] Z. Zhao , K. Bao , D. Li , D. Duan , F. Tian , X. Jin , C. Chen , X. Huang , B. Liu , T. Cui , Sci. Rep. 2014, 4, 4797. 24762713 10.1038/srep04797 PMC3999448

[24] U. Müller , Inorganic Structural Chemistry, Wiley, Chichester, 2006.

[25] L. Paseta , G. Potier , S. Sorribas , J. Coronas , ACS Sustainable Chem. Eng. 2016, 4, 3780–3785.

[26] C. S. Cundy , P. A. Cox , Microporous Mesoporous Mater. 2005, 82, 1–78.

[27] H. Alkhaldi , P. Kroll , J. Phys. Chem. C 2019, 123, 7054–7060.

[28] I. E. Collings , E. Bykova , M. Bykov , S. Petitgirard , M. Hanfland , D. Paliwoda , L. Dubrovinsky , N. Dubrovinskaia , ChemPhysChem 2016, 17, 3369–3372. 27500946 10.1002/cphc.201600854

[29] I. E. Collings , A. L. Goodwin , J. Appl. Phys. 2019, 126, 181101.

[30] C. L. Hobday , C. H. Woodall , M. J. Lennox , M. Frost , K. Kamenev , T. Düren , C. A. Morrison , S. A. Moggach , Nat. Commun. 2018, 9, 1429. 29650966 10.1038/s41467-018-03878-6 PMC5897325

[31] A. F. Young , C. Sanloup , E. Gregoryanz , S. Scandolo , R. J. Hemley , H. Mao , Phys. Rev. Lett. 2006, 96, 155501. 16712167 10.1103/PhysRevLett.96.155501

[32] D. Pfister , K. Schäfer , C. Ott , B. Gerke , R. Pöttgen , O. Janka , M. Baumgartner , A. Efimova , A. Hohmann , P. Schmidt , et al., Adv. Mater. 2016, 28, 9783–9791. 27624093 10.1002/adma.201603135

[33] V. Soghomonian , Q. Chen , R. C. Haushalter , J. Zubieta , C. J. O'Connor , Science 1993, 259, 1596–1599. 17733025 10.1126/science.259.5101.1596

[34] J. Binns , M. E. Donnelly , M. Pena-Alvarez , M. Wang , E. Gregoryanz , A. Hermann , P. Dalladay-Simpson , R. T. Howie , J. Phys. Chem. Lett. 2019, 10, 1109–1114. 30785288 10.1021/acs.jpclett.9b00070

[35] A. B. Mei , O. Hellman , N. Wireklint , C. M. Schlepütz , D. G. Sangiovanni , B. Alling , A. Rockett , L. Hultman , I. Petrov , J. E. Greene , Phys. Rev. B 2015, 91, 054101.

[36] A. Zerr , G. Miehe , R. Riedel , Nat. Mater. 2003, 2, 185–189. 12612677 10.1038/nmat836

