# Supplementary material for: High‐Pressure Synthesis of Metal–Inorganic Frameworks Hf4N20⋅N2, WN8⋅N2, and Os5N28⋅3 N2 with Polymeric Nitrogen Linkers
Source: Angew Chem Int Ed Engl. 2020 May 8;59(26):10321–6. doi: 10.1002/anie.202002487 (PMC7317814; doi:10.1002/anie.202002487)
Supplement: Supplementary file 1 — Supplementary [file ANIE-59-10321-s001.pdf]

Angewandte Chemie
Eine Zeitschrift der Gesellschaft Deutscher Chemiker

Supporting Information

High-Pressure Synthesis of Metal–Inorganic Frameworks $Hf_4N_{20}{\cdot}N_2$, $WN_8{\cdot}N_2$, and $Os_5N_{28}{\cdot}3\,N_2$ with Polymeric Nitrogen Linkers

Maxim Bykov, Stella Chariton, Elena Bykova, Saiana Khandarkhaeva, Timofey Fedotenko, Alena V. Ponomareva, Johan Tidholm, Ferenc Tasnádi, Igor A. Abrikosov, Pavel Sedmak, Vitali Prakapenka, Michael Hanfland, Hanns-Peter Liermann, Mohammad Mahmood, Alexander F. Goncharov, Natalia Dubrovinskaia, and Leonid Dubrovinsky*

anie_202002487_sm_miscellaneous_information.pdf

Table of Contents

Experimental Procedures 2
Crystallographic structure details 3
Phonon spectra 6
Powder XRD of decompressed Os-N compound 8
References 8

Experimental Procedures

High-pressure synthesis

For the high-pressure experiments Hf, Os and W metals were loaded in BX90-type diamond anvil cells[1] with Boehler-Almax type diamonds[2] (culet size 120 μm). Rhenium gaskets were pre-indented from an initial thickness of 200 μm down to 25 μm and laser-drilled to create circular pressure chambers of 60 μm in diameter. The DACs were loaded with nitrogen (initial pressure of 1.7 kbar) that served as a reagent and as a pressure-transmitting medium. Hf, W, and Os samples were compressed to a pressure about 105 GPa and laser-heated to 1900(200), 2700 (200) and 2800(150) K respectively.

X-ray diffraction

The reaction products contained multiple good-quality single-crystalline domains of novel phases and they were studied by synchrotron single-crystal X-ray diffraction at the beamlines P02.2 (PetraIII, DESY, Hamburg, Germany Hf sample), GSECARS 13IDD (APS, Argonne, USA, W sample), ID15b (Grenoble, France, W sample) and ID11 (Grenoble, France, Os samples). The following beamline setups were used. P02.2: λ = 0.29 Å, beam size ~2×2 μm^2, Perkin Elmer XRD 1621 detector; ID15B: λ = 0.41, beam size ~10×10 μm^2, Mar555 flat panel detector; GSECARS: λ = 0.2952 Å, beam size ~3×3 μm^2, Pilatus CdTe 1M detector. For the single-crystal XRD measurements samples were rotated around a vertical ω-axis in a range ±38°. The diffraction images were collected with an angular step Δω = 0.5° and an exposure time of 1s/frame. For analysis of the single-crystal diffraction data (indexing, data integration, frame scaling and absorption correction) we used the *CrysAlis^Pro^* software package. To calibrate an instrumental model in the *CrysAlis^Pro^* software, *i.e.*, the sample-to-detector distance, detector's origin, offsets of goniometer angles, and rotation of both X-ray beam and the detector around the instrument axis, we used a single crystal of orthoenstatite ($(Mg_{1.93}Fe_{0.06})(Si_{1.93}, Al_{0.06})O_6$, *Pbca* space group, *a* = 8.8117(2), *b* = 5.18320(10), and *c* = 18.2391(3) Å). The same calibration crystal was used at all the beamlines. Powder diffraction measurements were performed either without sample rotation (still images) or upon continuous rotation in the range ±20°ω. The images were integrated to powder patterns with DIOPTAS software[3]. Le-Bail fits of the diffraction patterns were performed with the Jana2006 software.

Structure solution and refinement

The structure was solved with the ShelXT structure solution program[4] using intrinsic phasing and refined with the Jana 2006 program[5]. CSD 1978265-1978268 contain the supplementary crystallographic data for this paper. These data can be obtained free of charge from FIZ Karlsruhe *via* www.ccdc.cam.ac.uk/structures

Theoretical calculations

For *ab initio* electronic structure calculations of MIFs the projector-augmented-wave (PAW) method [6] as implemented in the VASP code was employed [7–9]. For calculation of exchange–correlation energy we used the Perdew-Burke-Ernzerhof GGA (PBE)[10] and non-empirical strongly constrained and appropriately normed (SCAN) meta-GGA [11]. Note that the latter are significantly more time consuming as compared to the former. Convergence has been achieved with 700 eV energy cutoff for the plane wave basis and a ((8×06×17), (19×13×13), (7×7×26)) Monkhorst–Pack k-points [12] type sampling of the Brillouin zone for $Hf_4N_{20}\cdot N_2$, $WN_8\cdot N_2$ and $Os_5N_{28}\cdot 3N_2$, respectively. Gaussian smearing technique was chosen with smearing of 0.10 eV. The convergence criterion for the electronic subsystem has been chosen to be equal to 10^{-5} eV for two subsequent iterations, and the ionic relaxation loop within the conjugated gradient method was stopped when forces became of the order of 10^{-4} eV/Å.

First, we establish the reliability of theoretical simulations and select approximations that optimize accuracy and efficiency of the simulations. At this point crystal structure for each of the experimentally synthesized material was fully optimized at fixed volumes (corresponding to experimentally measured volumes for respective system), including the optimization of atomic positions and the shapes of the unit cells. Then we carried out calculations at various volumes and determined parameters of the equilibrium state by fitting energy-volume relations with polynomial form of equation of state. For $WN_8 \cdot N_2$ and $Os_5N_{28} \cdot 3N_2$ good agreement with the experimental structural characteristics was obtained already in simulations with PBE functional (Table S2), and the use of the SCAN functional did not modify them significantly. However, in the case of $Hf_4N_{20} \cdot N_2$ the agreement with the experiment was significantly improved using the SCAN functional (Table S2). The lattice parameters obtained using PBE are in worse agreement with experiment, which may be a consequence of high number of various nitrogen units and the different types of atomic bonds represented in the compound. In Ref. [13] SCAN has been tested in diversely bonded systems, where it was shown to be sophisticated enough to model a wide range of physical structures without being fitted to any bonded system. Inclusion of electronic correlations through the on-site Hubbard interaction with U=2 eV and J=1 eV in the Dudarev's approach [11] further improves the agreement between theory and experiment (Table S1). Thus, in the main text we present the theoretical results calculated with SCAN + U for $Hf_4N_{20} \cdot N_2$ (U =2eV, J=1 eV) and with PBE functional for $WN_8 \cdot N_2$ and $Os_5N_{28} \cdot 3N_2$.

Simulations of different occupancies of nitrogen molecules in the channels in Os-N were carried out using 304-atom ($Os_5N_{28} \cdot 2.5N_2$), 78 and 156 -atom ($Os_5N_{28} \cdot 3N_2$), and 164-atom periodic ($Os_5N_{28} \cdot 4N_2$) cells. The relaxation of the $Os_5N_{28} \cdot xN_2$ structure on the synthesis volume with empty channels of both types showed that the structure begun to collapse with a strong change in the shape of both channels, while the calculated pressure for relaxed structure was two times lower than the experimental one. When filling only rectangular channels, a strong distortion of the structure and a mismatch between the calculated pressure and the synthesis value were observed as well. Our calculations showed that the concentration x = 3 in $Os_5N_{28} \cdot xN_2$ gave the best fit to the experimentally determined structure. For x > 3 nitrogen begun to form chains in the octagonal channel, at x ≤ 3 nitrogen was in a molecular state in both rectangular and octagonal-shaped channels, but the pressure and structure did not correspond to the experimental observations.

Phonon dispersion relations were calculated in harmonic approximation at 0 K using the finite displacement approach implemented in Phonopy [13]. For Hf_2N_{11} converged phonon dispersion relations were achieved using a (2×2×2) supercell, for WN_8 N_2 a (3×3×3) and for Os_5N_{28} $3N_2$ a (1×1×4) sized supercell had to be applied. In all these cases the (5×5×5) Monkhorst-Pack [11] k-point sampling was used to ensure accuracy of the interatomic forces. In case of Hf_4N_{20} N_2 the high memory computational demand of the SCAN and SCAN+U approximations together with the required 700 eV energy cutoff parameter made calculations prohibitively time-consuming. Therefore, the largest supercell for which we obtained results with (5×5×5) Monkhorst-Pack k-point sampling had a size of (2×2×2). Figures 1-4 show the calculated phonon dispersion relations at 0 GPa and various pressures including one close to the experimental pressure from Table S1.

Crystallographic structure details

Table S1. Selected crystal structure details of $Hf_4N_{20} \cdot N_2$, $WN_8 \cdot N_2$ and $Os_5N_{28} \cdot 3N_2$ obtained in our experiments. Full crystallographic information is given in supplementary crystallographic information files.

	**$Hf_4N_{20} \cdot N_2$**	**$WN_8 \cdot N_2$**	**$Os_5N_{28} \cdot 3N_2$**
Pressure, GPa	105	105	106
Symmetry	*Cmmm*	*Immm*	*Pnnm*
Z	2	2	2
Lattice parameters, Å	*a* = 8.5695(13),<br>*b* = 11.855(18),<br>*c* = 3.6042(10)	*a* = 3.4978(6),<br>*b*= 6.245(2),<br>*c* = 6.5043(12)	*a* = 12.1054(10),<br>*b* = 12.3071(6),<br>*c* = 3.4330(6)
Unit cell volume, Å^3	366.16(13)	142.07(6)	511.46(10)
R_1/wR_2	0.0773/0.0996	0.0488/0.1184	0.0509/0.0634
Atomic coordinates	Hf1 (0.5 0.67584 0.0)<br>Hf2 (0.31374, 0.5, 0.5)<br>N1 (0.5, 0.6123, 0.5)<br>N2 (0.365, 0.789, 0.681)<br>N3 (0.276, 0.8664, 0.81)<br>N4 (0.577, 0.5, 0.0)<br>N5 (0.0, 0.5, 0.86)	W (0 0 0)<br>N1 (0.316, 0.184, -0.182)<br>N2 (0.179, 0.5, 0)	Os1 (0.5 0.5 0.5)<br>Os2 (0.24670, 0.43584, 0.5)<br>Os3 (0.56778, 0.74842, 0.5)<br>N1 (0.36370, 0.49780, 0.177)<br>N2 (0.45550, 0.81640, 0.191)<br>N3 (0.1828, 0.5509, 0.183)<br>N4 (0.3173, 0.323, 0.194)<br>N5 (0.3691, 0.8672, 0.311)<br>N6 (0.501, 0.6324, 0.184)

			N7 (0.1337, 0.6367, 0.31)<br>N8 (0.5, 0, 0.155)<br>N9 (0.199, 0.191, 0.66)

Table S2. Theoretical structural parameters of $Hf_4N_{20}{\cdot}N_2$, $WN_8{\cdot}N_2$ and $Os_5N_{28}{\cdot}3N_2$ obtained using different exchange-correlation functionals. Note that volumes of the unit cells were fixed in theoretical simulations, while pressure, lattice parameters and equilibrium state parameters were calculated.

	$Hf_4N_{20}{\cdot}N_2$			$WN_8{\cdot}N_2$		$Os_5N_{28}{\cdot}3N_2$	
	SCAN	SCAN + U	PBE	SCAN	PBE	SCAN	PBE
Pressure, GPa	93	96.4	94	100	103	102	108
Lattice parameters, Å	a=8.511<br>b=11.758<br>c =3.659	a= 8.515<br>b= 11.804<br>c= 3.643	a= 8.323<br>b= 11.968<br>c= 3.676	a= 3.502<br>b= 6.186<br>c= 6.558	a= 3.511<br>b= 6.156<br>c= 6.572	a= 12.071<br>b= 12.266<br>c= 3.459	a= 12.041<br>b= 12.255<br>c= 3.4704
Volume, $Å^3$	366.16	366.16	366.16	142.07	142.07	512.12	512.12
Equation of state parameters							
V_0, $Å^3$	494	497.9	509	184	188	670	670
K_0, GPa	169	164.6	147	204	177	180	180
K'	5.7	5.07	6.2	6.2	7.2	7.3	7.3

Table S3. Refinement and crystal structure details of Hf_2N_{11} (compound **4**) at 105 GPa.

Chemical formula	$HfN_{5.5}$
Crystal data	
M_r	255.5
Crystal system, space group	Hexagonal, $P6_422$
Pressure (GPa)	105
a, *c* (Å)	6.3153 (7), 7.3916 (11)
V ($Å^3$)	255.31 (6)
Z	6
Radiation type	Synchrotron, λ = 0.2892 Å
λ (mm^{-1})	5.76
**Data collection**	
Diffractometer	LH@P02.2
Absorption correction	Multi-scan<br>*CrysAlis PRO* 1.171.39.46 (Rigaku Oxford Diffraction, 2018) Empirical absorption correction using spherical harmonics, implemented in SCALE3 ABSPACK scaling algorithm.
No. of measured, independent and observed [*I* > 3□(*I*)] reflections	1599, 560, 506
R_{int}	0.071
$(\sin θ/λ)_{max}$ ($Å^{-1}$)	1.077
**Refinement**	
$R[F^2 > 2σ(F^2)]$, $wR(F^2)$, S	0.048, 0.101, 1.71
No. of reflections	560
No. of parameters	17
$Δρ_{max}$, $Δρ_{min}$ (e $Å^{-3}$)	7.70, −4.32
Absolute structure	180 of Friedel pairs used in the refinement
**Atomic coordinates**	
Hf1	(0.20558(6) 0.79442(6) 0.666667)
N1	(0.5 0 0.5)
N2	(0.0939(18) 0 0.5)

N3	(0.5451(16) 0.8244(17) 0.7522(11))
N4	(0.6170(18) 0.7217(18) 0.6313(11))

Table S4. Theoretical structural parameters of Hf_2N_{11} (compound 4).

	PBE
Pressure, GPa	105
Lattice parameters, Å	a= 6.3196<br>b= 6.3196<br>c= 7.3815
Volume, Å^3	255.3
Equation of state	V_0 = 339.7 Å^3<br>K_0 = 193 GPa<br>K' = 5.7

Table S5. Refinement and crystal structure details of Hf_3N_4 at 73 GPa

Chemical formula	Hf_3N_4
Crystal data	
M_r	591.5
Crystal system, space group	Cubic, $I\bar{4}3d$
Pressure (GPa)	73
a (Å)	6.2946
V (Å^3)	249.41
Z	4
Radiation type	X-ray, λ = 0.2952 Å
λ (mm^{-1})	3.07
**Data collection**	
Diffractometer	13IDD @ APS
Absorption correction	Multi-Scan
No. of measured, independent and observed [$I > 3\sigma(I)$] reflections	311, 83, 78
R_{int}	0.164
$(\sin \theta/\lambda)_{max}$ (Å^{-1})	0.877
**Refinement**	
$R[F^2 > 2\sigma(F^2)]$, $wR(F^2)$, S	0.060, 0.077, 5.78
No. of reflections	83
No. of parameters	6
$\Delta\rho_{max}$, $\Delta\rho_{min}$ (e Å^{-3})	8.10, -6.62
Absolute structure	32 of Friedel pairs used in the refinement
**Atomic coordinates**	
Hf	12*a* (3/8, 0, 1/4)
N	16*c* (0.159, 0.159, 0.159)

Phonon spectra

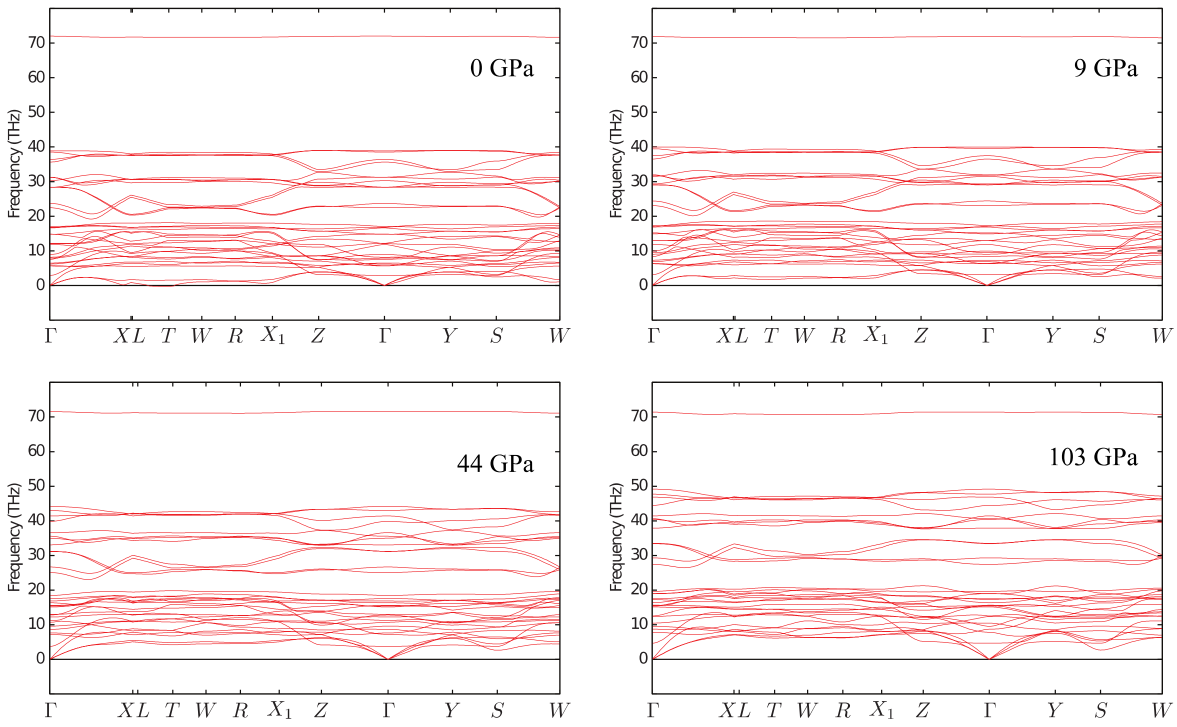

Figure S1. Phonon dispersion relations for WN_8 N_2 (compound **2**) at various pressures.

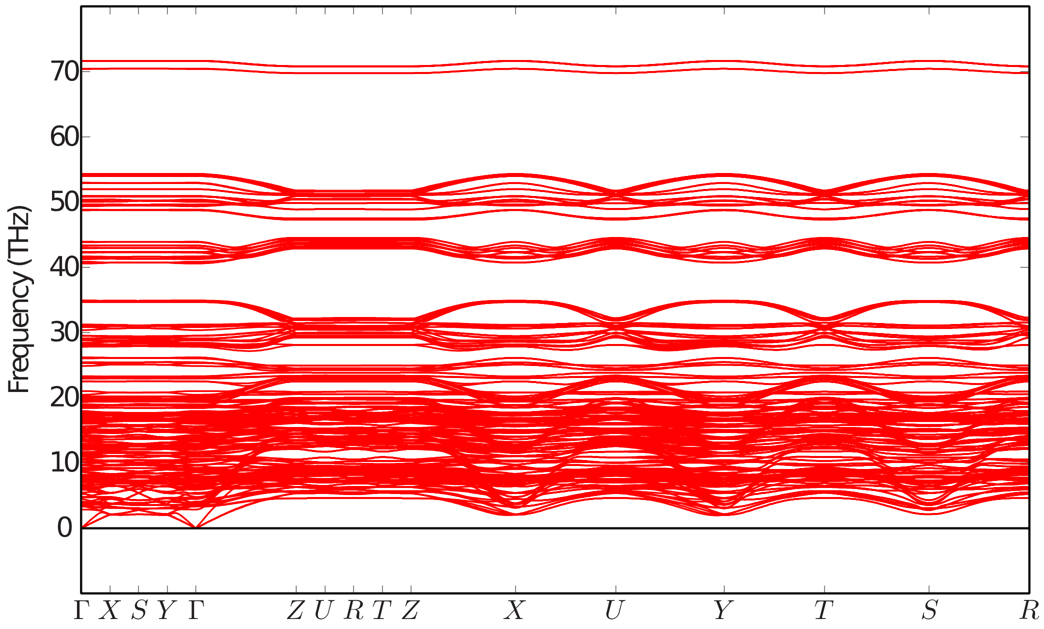

Figure S2. Phonon dispersion relations for Os_5N_{28} $3N_2$ (compound **3**) at pressure P=100 GPa.

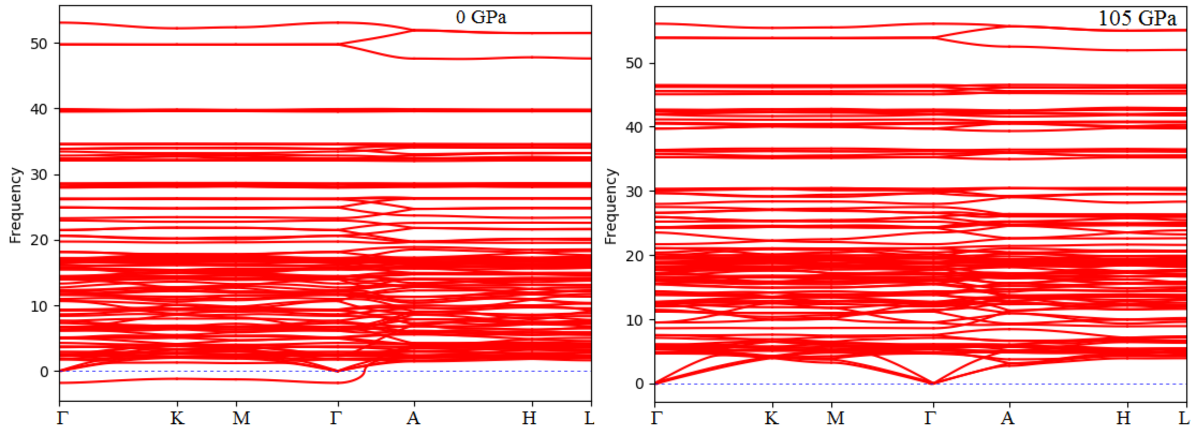

Figure S3. Phonon dispersion relations for Hf_2N_{11} (compound **4**) at p=0 GPa and p=105 GPa.

a)

SCAN

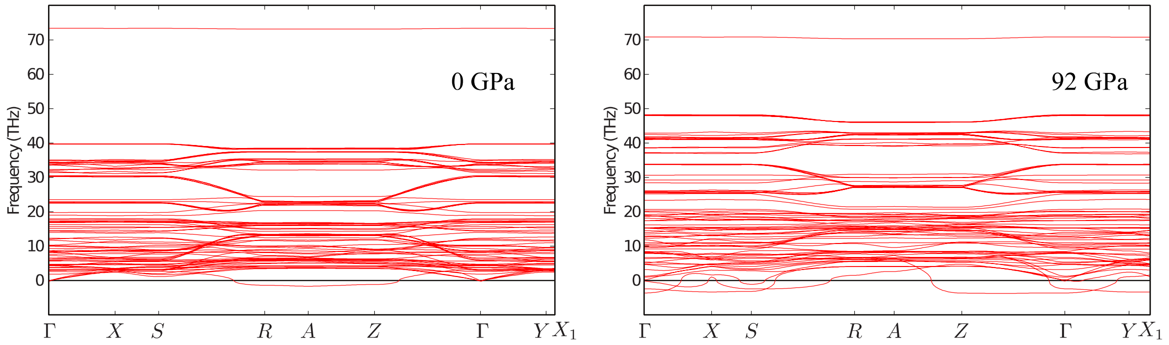

b)

SCAN+U

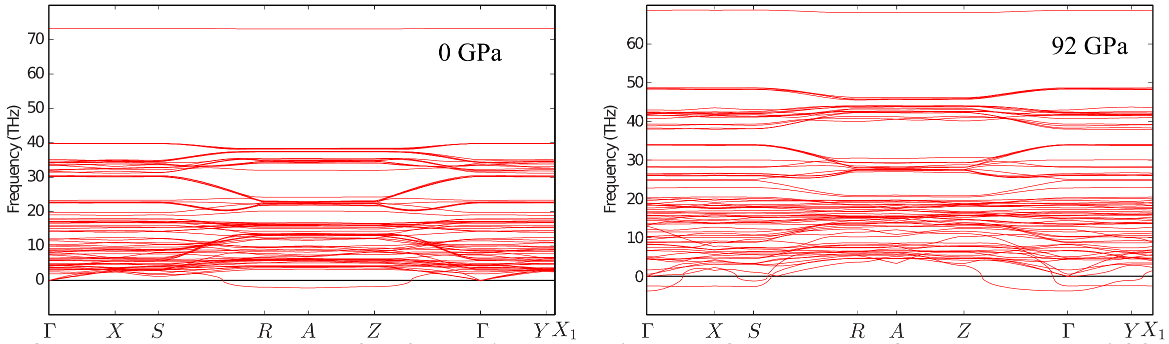

Figure S4. Phonon dispersion relations for $Hf_4N_{20}{\cdot}N_2$ (compound **1**) at P = 0 GPa and P = 92 GPa calculated with a) SCAN functional and b) SCAN+U functional with U=2 eV, J=1 eV.

Powder XRD of decompressed Os-N compound

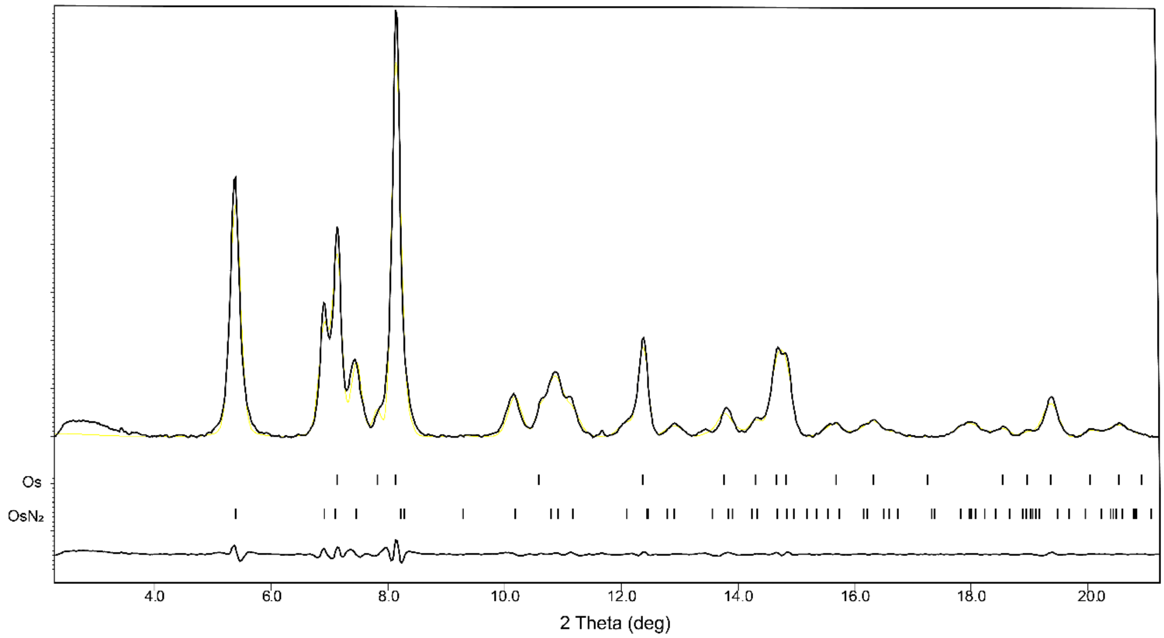

Figure S5. Powder diffraction pattern of the decompressed Os-N sample (λ = 0.2952 Å).

References

[1] I. Kantor, V. Prakapenka, A. Kantor, P. Dera, A. Kurnosov, S. Sinogeikin, N. Dubrovinskaia, L. Dubrovinsky, *Rev. Sci. Instrum.* **2012**, *83*, 125102.

[2] R. Boehler, *Rev. Sci. Instrum.* **2006**, *77*, 115103.

[3] C. Prescher, V. B. Prakapenka, *High Press. Res.* **2015**, *35*, 223–230.

[4] G. M. Sheldrick, *Acta Crystallogr. Sect. A Found. Adv.* **2015**, *71*, 3–8.

[5] V. Petříček, M. Dušek, J. Plášil, *Zeitschrift für Krist. - Cryst. Mater.* **2016**, *231*, 583–599.

[6] P. E. Blöchl, *Phys. Rev. B* **1994**, *50*, 17953–17979.

[7] G. Kresse, J. Furthmüller, *Phys. Rev. B* **1996**, *54*, 11169–11186.

[8] G. Kresse, J. Furthmüller, *Comput. Mater. Sci.* **1996**, *6*, 15–50.

[9] G. Kresse, D. Joubert, *Phys. Rev. B* **1999**, *59*, 1758–1775.

[10] J. P. Perdew, K. Burke, M. Ernzerhof, *Phys. Rev. Lett.* **1996**, *77*, 3865–3868.

[11] J. Sun, A. Ruzsinszky, J. Perdew, *Phys. Rev. Lett.* **2015**, *115*, 1–6.

[12] H. J. Monkhorst, J. D. Pack, *Phys. Rev. B* **1976**, *13*, 5188–5192.

[13] J. Sun, R. C. Remsing, Y. Zhang, Z. Sun, A. Ruzsinszky, H. Peng, Z. Yang, A. Paul, U. Waghmare, X. Wu, et al., *Nat. Chem.* **2016**, *8*, 831–836.
